# *QuickStats:* Age-Adjusted Death Rates* for Alzheimer Disease^^†^^ Among Adults Aged ≥65 Years, by Sex and Race/Hispanic Origin^^§^^ — National Vital Statistics System, 2018

**DOI:** 10.15585/mmwr.mm6944a7

**Published:** 2020-11-06

**Figure:**
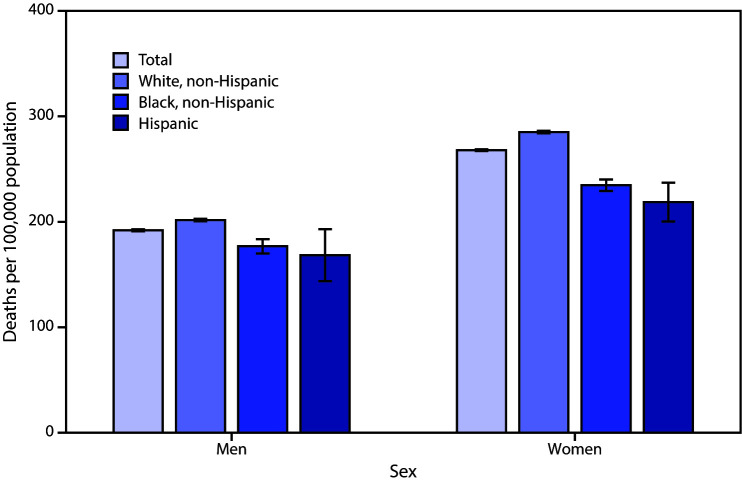
In 2018, the age-adjusted death rate for Alzheimer disease among adults aged ≥65 years was higher for women (267.9 deaths per 100,000) than for men (191.9). Among men, non-Hispanic White men had the highest death rate (201.7) compared with non-Hispanic Black (176.8) and Hispanic (168.4) men. Among women, non-Hispanic White women (285.1) had the highest death rate, followed by non-Hispanic Black (234.7) and Hispanic (218.8) women. Compared with men, women had higher age-adjusted death rates from Alzheimer disease in all three race and Hispanic-origin groups.

**Source:** National Center for Health Statistics, National Vital Statistics System, Mortality Data. https://www.cdc.gov/nchs/deaths.htm.

